# Metal Exposure and SNCA rs356219 Polymorphism Associated With Parkinson Disease and Parkinsonism

**DOI:** 10.3389/fneur.2020.556337

**Published:** 2020-12-09

**Authors:** Roberto G. Lucchini, Stefano Guazzetti, Stefano Renzetti, Karin Broberg, Margherita Caci, Loredana Covolo, Patrizia Crippa, Umberto Gelatti, Dana Hashim, Manuela Oppini, Fulvio Pepe, Andrea Pilotto, Chiara Passeri, Donatella Placidi, Maira Cristina Rizzetti, Marinella Turla, Karin Wahlberg, Alessandro Padovani

**Affiliations:** ^1^Robert Stempel College of Public Health, Florida International University, Miami, FL, United States; ^2^Department of Medical Surgical Specialities, Radiological Sciences and Public Health, University of Brescia, Brescia, Italy; ^3^Azienda USL-IRCCS di Reggio Emilia, Reggio Emilia, Italy; ^4^Division of Occupational and Environmental Medicine, Lund University, Lund, Sweden; ^5^Institute of Environmental Medicine, Karolinska Institutet, Solna, Sweden; ^6^Ancelle Della Carità, Brescia, Italy; ^7^Hematology & Medical Oncology, Icahn School of Medicine at Mount Sinai, New York, NY, United States; ^8^Neurology, Poliambulanza Foundation, Brescia, Italy; ^9^Neurology Unit, Department of Clinical and Experimental Sciences, University of Brescia, Brescia, Italy; ^10^Parkinson Rehabilitation Center, Ospedale S. Isidoro - FERB Onlus, Trescore Balneario, Bergamo, Italy; ^11^Esine Hospital of Valcamonica, Brescia, Italy

**Keywords:** metal exposure, SNCA gene, parkinsonism, parkinson disease, occupational exposure, manganese

## Abstract

**Objective:** In the province of Brescia, Italy, historical neurotoxic metal exposure has occurred for several decades. This study aimed to explore the role of metal exposure and genetics on Parkinson's Disease (PD) and Parkinsonism.

**Methods:** Cases were enrolled from four local clinics for movement disorders. Randomly selected controls non-affected by neurological or psychiatric conditions were enrolled from the same health centers keeping a similar gender ratio and age distribution as for cases. Data on sociodemographic variables, clinical onset and life habits were collected besides accurate occupational and residential history. Blood samples were collected from all participants for genotyping of target polymorphisms in genes linked to PD and/or metal transport.

**Results:** A total number of 432 cases and 444 controls were enrolled in the study, with average age of 71 years (72.2 for cases and 70 for controls). The average age at diagnosis was 65.9 years (SD 9.9). Among the potential risk factors, family history of PD or Parkinsonism showed the strongest association with the diseases (OR = 4.2, 95% CI 2.3, 7.6 on PD; OR = 4.3, 95% CI 1.9, 9.5 for Parkinsonism), followed by polymorphism rs356219 in the alpha-synuclein (*SNCA*) gene (OR = 2.03, 95% CI 1.3, 3.3 for CC vs. TT on PD; OR = 2.5, 95% CI 1.1, 5.3 for CC vs. TT on Parkinsonism), exposure to metals (OR = 2.4;, 95% CI 1.3, 4.2 on PD), being born in a farm (OR = 1.8; 95% CI 1.1, 2.8 on PD; OR = 2.6; 95% CI 1.4, 4.9 on Parkinsonism) and being born in the province of Brescia (OR = 1.7; 95% CI 1.0, 2.9 on PD). Conditional OR of having PD depending by *SNCA* polymorphism and metal exposure highlights higher risk of PD among CC *SNCA* carriers and being exposed to metals. However, the interaction term was not statistically significant.

**Conclusions:** Lifetime exposure to metals and genetic variation in *SNCA* gene are relevant determinants of PD and Parkinsonism in the highly industrialized area of Brescia, Italy. The lack of evidence of statistical interaction between environmental and genetic factors may be due to the low frequencies of subjects representing the exposure categories and the polymorphism variants and does not rule out the biological interaction.

## Introduction

The province of Brescia, northern Italy, was characterized by an historical metallurgic and ferromanganese industrial activities. A higher prevalence of Parkinsonism has been observed among municipalities closer to ferromanganese plants (492/100,000) compared to other province municipalities (321/100,000) (1). A significant association between the Bayesian Standardized Morbidity Ratios (SMRs) for Parkinsonism and dust manganese (Mn) concentrations was also found (1). A case-control study in the same area showed higher levels of blood Mn, copper (Cu) and zinc (Zn) among the cases residing around the ferroalloy plants of Valcamonica (2–4). Studies from our group have shown high levels of Mn, arsenic (As), cadmium (Cd), chromium (Cr), Cu, iron (Fe), lead (Pb), nickel (Ni), Zn (5–9) in environmental media including airborne particles (5, 10–13), indoor and outdoor deposited dust (7), attic dust (6), soil (8, 14), and locally grown produce (9). This exposure to metals has been associated with biomarkers (15) and neurobehavioral outcomes in children (16–19), workers (20–22), and elderly (23, 24) residing in the area, indicating adverse health outcomes of the metal exposure. Further, genetic susceptibility for some of the metal-associated effects have been observed (25–29). Essential metals including Zn, Cu, Mn, and Fe play critical roles in the pathophysiology of neurodegenerative diseases including PD (30), also by inducing alpha-synuclein modifications (31–34).

Gene-environment interactions have been identified in association with PD (35), particularly for metal exposures (36). Measuring genetic susceptibility markers in metal exposed populations (37) becomes critical to identify susceptible groups to Parkinsonian disturbances and preventing disease onset and progression. In a case-control study of 874 individuals we aimed to assess whether PD is associated with metal exposure, mainly assessed through job exposure, and examine the associations between PD- and metal-associated single nucleotide polymorphisms (SNPs) and its interaction with metal exposure.

## Methods

### Case Definition and Enrollment

The case definition was not restricted to the diagnostic criteria of IPD, but extended to the broader classification of Parkinsonism (i.e., Parkinsonism and/or PD). This was defined by the presence of at least two of the cardinal features: (i) bradykinesia/akinesia; (ii) rigidity; (iii) tremor; (iv) postural instability (38), and with the exclusion of cases based on vascular, iatrogenic, and traumatic origin. Four centers followed a common case definition and diagnostic protocol and prevalent cases were recruited and reviewed by a single neurologist from any one of the centers specialized in movement disorders located in the Province of Brescia at the following Institutions: ASST-Spedali Civili of Brescia, Esine Hospital of Valcamonica, Poliambulanza Foundation, Ancelle Domus Salutis. Each patient was evaluated by the neurologists attending in each center: authors AP, MCR, AP at Spedali civili, FP at Poliambulanza Foundation, MT at Esine Hospital, PC at Ancelle Brescia. Diagnosis of PD and atypical parkinsonism was performed according to clinical criteria (39). Non-Parkinsonian/PD controls were recruited from the clinics of Dermatology, Orthopedics, Ophthalmology and Otorhinolaryngology of the same hospitals, in order to provide a similar geographic distribution of the residencies of the cases.

All subjects underwent blood sampling for DNA analyses at enrollment.

### Standard Protocol Approvals, Registrations, and Patient Consents

The study design and consent were approved by the Ethical Committee of the Department of Health, Brescia, and each participant was consented after receiving written and oral explanation about the study aims and methodology. Exclusion criteria were any neurological or psychiatric condition other than parkinsonian disorders.

### Questionnaires

Data on demographics, lifestyle habits, family history of Parkinsonism/PD (if either the mother or the father had Parkinsonism or PD; only parents were considered to decrease the potential recall bias) and, for the cases, clinical diagnosis, age at onset and clinical data were collected through questionnaires. An exposure questionnaire was administered to cases and controls to gain information about their occupational and residential history. Self-reported occupational exposures to specific neurotoxic metals (aluminum, antimony, As, beryllium, Cd, Cu, Cr, gallium, magnesium, Mn, mercury, Ni, Pb) were recorded. Because of the low frequencies of the exposure to the single metals, an overall exposure measure was considered in the study identifying those subjects with at least one occupational exposure to metals in their job life (before diagnosis for the cases).

Participants were also asked to report job titles, tasks, industries, starting dates and duration (in years) of each job.

### Genotyping

Polymorphisms known to be associated with Parkinsonism and/or PD and/or with handling (e.g., uptake and transport) of metals in the body were measured: (i) solute carrier family 30 member 10 (*SLC30A10*: rs2275707, rs12064812, rs1776029) (27); (ii) solute carrier family 39 member 8 (*SLC39A8/ZIP8*: rs13107325) (28); (iii) PARK9-associated cation-transporting ATPase 13A2 (*ATP13A2*: rs4920608 and rs2871776) (40); (iv) synuclein alpha (*SNCA*: rs356219) (41); (v) divalent metal transporter 1 (*DMT1*: IVS4+44G/T, rs224589) (42).

DNA was extracted from 0.2 ml of peripheral whole blood samples using the QIAamp DNA Blood Mini kit (Qiagen, Hilden, Germany). Genotyping was performed with TaqMan real time PCR for rs2275707, rs12064812 and rs13107325 (Thermo Scientific assays), as previously described (26, 27); and for rs4920608, rs2871776, rs224589, and rs356219 (Life Technologies assays). Rs1776029 is situated in the middle of an Alu-repeat region of approximately 300 bp in length, and a satisfactory TaqMan SNP assay with respect to specificity and amplicon length could not be designed for this SNP. Therefore, rs1776029 was instead genotyped by pyrosequencing as previously described (26). Generation of PCR products and pyrosequencing was performed using the PyroMark reagents and PSQ HS96 Pyrosequencing System (Qiagen) according to manufacturer's instructions. For quality control of genotyping data, >5% of samples were re-analyzed for all SNPs in a separate round of experiment with a 100% agreement between duplicate.

### Statistical Analyses

Hardy-Weinberg equilibrium (HWE) analysis was undertaken to determine deviation from genotype frequencies using the conventional χ2-test. The association of exposure to metals and each SNP with Parkinsonism and PD was assessed using multinomial regression setting controls as the reference category. For metals exposure, categories were defined as “Ever been exposed” vs. “Never exposed to.” For SNPs both χ2-test and multivariate (adjusted for all terms) odds ratios (ORs) were obtained as a measure of association between the genetic polymorphisms and the disease. The effects of the genetic polymorphisms were modeled assuming a co-dominant model, by considering the major homozygous as the reference level, the heterozygous as intermediate and the minor homozygous as the highest risk class (i.e., for the *SNCA* rs356219, creating an ordinal variable with three levels: TT, TC, CC, with the homozygote TT set as reference level).

Candidate variables were chosen based on the knowledge/hypotheses of risk factors and confounders for PD. The final adjusted model included variables for age, gender (male vs. female), smoking habits (ever/never), positive family history for PD or tremors, being born in a farm and being born in the province of Brescia (vs. outside the province of Brescia).

Age categories were defined as quartiles: (65,72], (72,78], (78,97], with reference quartile [40, 65]. We did not categorize age in decades because only 25 subjects were in the reference 40–50 class, and therefore the estimates of the contrasts with the baseline with the other strata would have resulted less accurate.

Kaplan Meier estimates and log-rank test were used to study the age at PD onset for the various *SNCA* rs356219 genotypes.

Analyses were performed using the R 3.6.1 (R Foundation for Statistical Computing).

## Results

Demographic characteristics and results from the interviews are displayed in Table 1. The number of enrolled cases and controls were, respectively, 432 and 444, yielding a total population of 876 subjects with participation rates of 75% for cases and 60% for controls. Most subjects were able to participate in the interviews alone, whereas 173 subjects (20% of the total), including 132 cases and 41 controls, participated with some degree of assistance by a caregiver. The overall mean age of subjects was 71 years (71.5 for cases with PD, 74.3 for cases with Parkinsonism and 70 for controls, *p* < 0.001). More than 10% of the cases were younger than 60 at visit day while mean age at diagnosis of PD and Parkinsonism was 65.9 (SD 9.9), and 7.5% were younger than 50 and still actively employed. Ten cases were employed in job sectors with potential exposure to neurotoxic agents such as metallurgy, agriculture and constructions, at the time of diagnosis (data not shown). The diagnosis included Idiopathic PD in 334 cases (77.3%, with average age at onset of 65.2 years), and Parkinsonism in 98 cases (22.7%, with average age at diagnosis of 68.3 years; Parkinsonism subgroups are reported in Table 1).

**Table 1:** Socio-demographics, lifestyle habits and exposures.

	**Controls (*N* = 444)**	**PD (*N* = 334)**	**Parkinsonism (*N* = 98)**	**Total (*N* = 876)**	***P*-value**
Sex					0.906
F	174 (39.2%)	126 (37.7%)	37 (37.8%)	337 (38.5%)	
M	270 (60.8%)	208 (62.3%)	61 (62.2%)	539 (61.5%)	
Age					<0.001
Mean (SD)	70.0 (9.8)	71.5 (9.9)	74.3 (7.5)	71.1 (9.7)	
**Age at diagnosis**
Mean (SD)		65.2 (10.2)	68.3 (8.5)	65.9 (9.9)	
**Age** **<** **50 at diagnosis**
No		294 (91.0%)	87 (97.8%)	381 (92.5%)	
Yes		29 (9.0%)	2 (2.2%)	31 (7.5%)	
Familiarity for PD or tremors					<0.001
No	393 (95.9%)	246 (83.4%)	73 (84.9%)	712 (90.0%)	
Yes	17 (4.1%)	49 (16.6%)	13 (15.1%)	79 (10.0%)	
Smoking habits					0.198
Ever	204 (46.2%)	133 (39.9%)	40 (40.8%)	377 (43.2%)	
Never	238 (53.8%)	200 (60.1%)	58 (59.2%)	496 (56.8%)	
Exposure to metals					0.001
No	404 (94.0%)	283 (86.0%)	87 (90.6%)	774 (90.5%)	
Yes	26 (6.0%)	46 (14.0%)	9 (9.4%)	81 (9.5%)	
Born in the province of Brescia					0.001
No	68 (15.5%)	29 (8.8%)	5 (5.1%)	102 (11.8%)	
Yes	370 (84.5%)	302 (91.2%)	93 (94.9%)	765 (88.2%)	
Born in a farmhouse					0.003
No	386 (88.3%)	267 (80.7%)	76 (77.6%)	729 (84.2%)	
Yes	51 (11.7%)	64 (19.3%)	22 (22.4%)	137 (15.8%)	
**Parkinsonisms**
Parkinson's disease dementia			35 (35.7%)	35 (35.7%)	
Cortico Basal Syndrome			12 (12.3%)	12 (12.3%)	
Dementia with Lewy bodies			11 (11.2%)	11 (11.2%)	
Progressive Supranuclear Palsy			9 (9.2%)	9 (9.2%)	
Multiple-system atrophy parkinsonian			5 (5.1%)	5 (5.1%)	
Multiple-system atrophy cerebellar dysfunction			1 (1.0%)	1 (1.0%)	
Unkown type			25 (25.5%)	25 (25.5%)	

*ANOVA and chi-squared test p-values are reported for continuous and categorical variable respectively*.

Regarding the subtypes of motor symptoms, 173 subjects (40% of cases) presented with the akinetic-rigid form of PD, 233 (53.9%) presented with the tremor-dominant PD, and 26 cases presented with a mixed phenotype.

Family history for PD or tremor, metal exposure, being born in a farmhouse and in the province of Brescia were more predominant among cases (both PD and Parkinsonism) than controls (all *P* < 0.01). Prevalence of subjects who ever smoked was higher among controls, confirming previous observations (43) but not statistically significant (*P* = 0.198).

We did not find deviations of Hardy-Weinberg equilibrium for any of the SNP studied here. Genotype frequencies of SNPs among cases and controls are reported in Table 2. Only *SNCA* rs356219 showed a marginally significant difference in genotype frequencies between PD, Parkinsonism cases and controls with higher frequencies of the rare allele (C) among cases. Also, in the multinomial regression, when adjusting for all covariates, the only significant SNP associated with PD and Parkinsonism was *SNCA* rs356219 (Table 3). Moreover, rs356219 CC carriers developed PD and Parkinsonism earlier when compared with the other subjects. The median age at the onset of PD and Parkinsonism was 64 years for the CC carriers and 68 for both the TC and TT (p at the Log-rank test 0.048) (Supplementary Figure 1).

**Table 2:** SNP frequency among cases and controls.

	**Controls (*N* = 444)**	**PD (*N* = 334)**	**Parkinsonism (*N* = 98)**	**Total (*N* = 876)**	***P*-value**
***SNCA*** **rs356219**					0.054
TT	181 (42.6%)	111 (34.5%)	30 (32.6%)	322 (38.4%)	
TC	190 (44.7%)	150 (46.6%)	46 (50.0%)	386 (46.0%)	
CC	54 (12.7%)	61 (18.9%)	16 (17.4%)	131 (15.6%)	
***SLC39A8*** **rs13107325**					0.989
CC	359 (85.1%)	276 (85.7%)	76 (85.4%)	711 (85.4%)	
CT	60 (14.2%)	44 (13.7%)	12 (13.5%)	116 (13.9%)	
TT	3 (0.7%)	2 (0.6%)	1 (1.1%)	6 (0.7%)	
***SLC30A10*** **rs12064812**					0.849
TT	206 (48.7%)	155 (48.1%)	44 (49.4%)	405 (48.6%)	
CT	172 (40.7%)	133 (41.3%)	39 (43.8%)	344 (41.2%)	
CC	45 (10.6%)	34 (10.6%)	6 (6.7%)	85 (10.2%)	
***SLC30A10*** **rs22757007**					0.796
AA	230 (54.5%)	178 (55.5%)	50 (56.2%)	458 (55.0%)	
AC	166 (39.3%)	117 (36.4%)	34 (38.2%)	317 (38.1%)	
CC	26 (6.2%)	26 (8.1%)	5 (5.6%)	57 (6.9%)	
***SLC30A10*** **rs1776029**					0.907
GG	257 (60.8%)	191 (59.3%)	54 (60.7%)	502 (60.2%)	
GA	143 (33.8%)	109 (33.9%)	31 (34.8%)	283 (33.9%)	
AA	23 (5.4%)	22 (6.8%)	4 (4.5%)	49 (5.9%)	
***ATP13A2*** **rs4920608**					0.114
TT	46 (33.6%)	52 (39.4%)	9 (22.0%)	107 (34.5%)	
TC	67 (48.9%)	66 (50.0%)	22 (53.7%)	155 (50.0%)	
CC	24 (17.5%)	14 (10.6%)	10 (24.4%)	48 (15.5%)	
***ATP13A2*** **rs2871776**					0.168
GG	122 (28.8%)	85 (26.0%)	37 (38.5%)	244 (28.8%)	
AG	206 (48.6%)	159 (48.6%)	42 (43.8%)	407 (48.1%)	
AA	96 (22.6%)	83 (25.4%)	17 (17.7%)	196 (23.1%)	
***DMT1*** **rs224589**					0.861
CC	248 (58.5%)	192 (58.7%)	51 (53.7%)	491 (58.0%)	
CA	142 (33.5%)	113 (34.6%)	36 (37.9%)	291 (34.4%)	
AA	34 (8.0%)	22 (6.7%)	8 (8.4%)	64 (7.6%)	

*Chi-squared test p-values are reported*.

**Table 3:** Results of multinomial regression analysis to test for the association between each polymorphism and PD and Parkinsonism adjusting for all the risk factors (age, gender, smoking status, PD or tremor familiarity, being born in a farm, being exposed to metals and being born in the province of Brescia).

	**PD**			**Parkinsonism**		
**Variables**	**OR**	**95% CI**	***P***	**OR**	**95% CI**	***P***
***SNCA*** **rs356219**
TC vs. TT	1.24	0.87–1.78	0.230	1.85	1.05–3.29	**0.035**
CC vs. TT	2.03	1.25–3.30	**0.004**	2.48	1.15–5.33	**0.020**
***SLC39A8*** **rs13107325**
CT vs. CC	1.01	0.64–1.61	0.956			
TT vs. CC	0.53	0.05–5.47	0.594			
***SLC30A10*** **rs12064812**
CT vs. TT	1.09	0.77–1.54	0.620	1.19	0.70–2.03	0.522
CC vs. TT	0.93	0.54–1.60	0.797	0.71	0.27–1.83	0.476
***SLC30A10*** **rs22757007**
AC vs. AA	0.93	0.66–1.31	0.673	0.92	0.54–1.57	0.765
CC vs. AA	0.95	0.49–1.83	0.875	0.45	0.13–1.62	0.223
***SLC30A10*** **rs1776029**
GA vs. GG	1.05	0.74–1.49	0.768	1.00	0.58–1.73	0.991
AA vs. GG	1.00	0.50 – 2.00	0.991	0.33	0.07–1.49	0.149
***ATP13A2*** **rs4920608**
TC vs. TT	0.91	0.50–1.67	0.768	2.44	0.88–6.74	0.086
CC vs. TT	0.45	0.18–1.13	0.088	3.29	0.96–11.28	0.058
***ATP13A2*** **rs2871776**
AG vs. GG	1.14	0.77–1.67	0.520	0.78	0.45–1.35	0.373
AA vs. GG	1.18	0.76–1.86	0.458	0.57	0.28–1.17	0.125
***DMT1*** **rs224589**
CA vs. CC	1.05	0.74–1.49	0.776	1.15	0.68–1.95	0.609
AA vs. CC	0.96	0.52–1.79	0.900	1.30	0.52–3.25	0.568

*Adjusted regression odds ratios, 95% CI, and their p-values are shown. For SLC39A8 rs13107325 only the association with PD is shown since carriers for the TT genotype were too few for Parkinsonism. p-values below 0.05 are shown in bold*.

**Figure 1:**
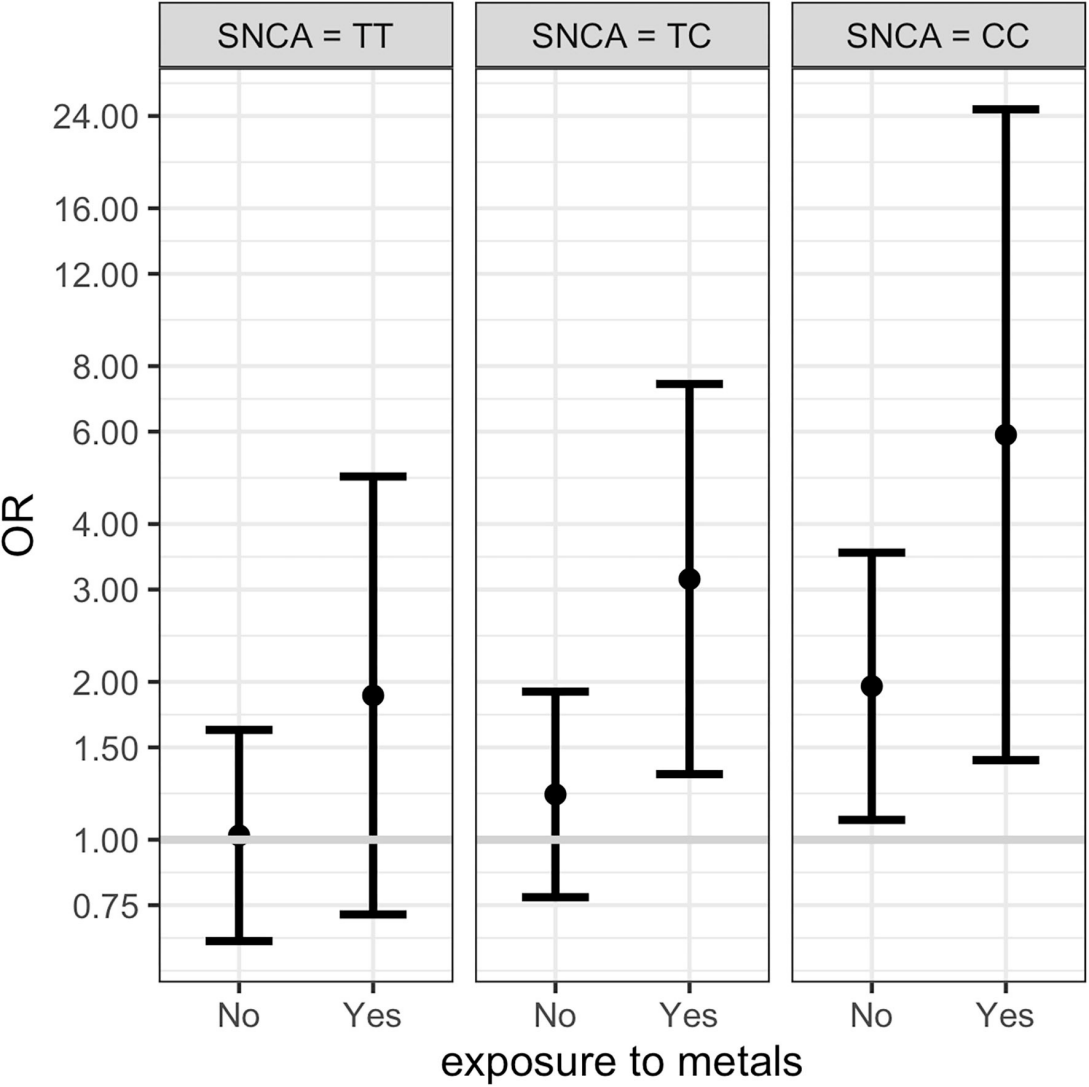
Conditional ORs of having PD depending by *SNCA* rs356219 and metal exposure (fixing the other covariates values at reference value) estimated from the multinomial regression. The first plot represents the conditional OR for subjects with SNCA TT polymorphism non-exposed and exposed to metals. The second and third columns shows the conditional ORs for TC and CC polymorphisms respectively. 95% CI are represented by the bars.

Results from multinomial regression analyses of risk factors vs. PD and Parkinsonism when considering *SNCA* rs356219 are reported in Table 4. We observed a significantly increased odds ratios for PD and Parkinsonism with family history of PD or tremor (OR = 4.23; OR = 4.3 respectively), *SNCA* rs356219 (OR = 2.03 CC vs. TT for PD; OR = 2.48 CC vs. TT, OR = 1.85 TC vs. TT for Parkinsonism), being exposed to metals (OR = 2.37 for PD), being born in a farm (OR = 1.77; OR = 2.63 for PD and Parkinsonism respectively) and being born in the province of Brescia (OR = 1.7 for PD). An interaction term between *SNCA* gene and metal exposure was added only when considering PD cases since we had too few observations in each category for Parkinsonism (among subjects with Parkinsonism and exposed to metals there were one CC, three TC and 5 TT *SNCA* carriers, respectively). Results showed there were increased OR for TC and CC carriers exposed to metals (Table 4). Figure 1 shows the conditional OR of having PD depending by *SNCA* polymorphism and metal exposure fixing the other covariates values at reference value highlighting higher risk of PD among CC *SNCA* carriers and being exposed to metals. However, the interaction term was not statistically significant.

**Table 4:** Results of multinomial and logistic regression analysis to test for the association between risk factors (age, gender, smoking status, PD or tremor familiarity, *SNCA* rs356219, being born in a farm, being exposed to metals and being born in the province of Brescia) and PD and Parkinsonism.

	**Multinomial regression**			**Logistic regression**		
**Variables**	**OR**	**95% CI**	***P***	**OR**	**95% CI**	***P***
**PD**						
Age (q.les) [65, 72] vs. [40, 65]	1.18	0.76–1.83	0.452	1.22	0.78–1.89	0.381
Age (q.les) [72, 78] vs. [40, 65]	1.45	0.92–2.30	0.112	1.45	0.91–2.30	0.118
Age (q.les) [78, 97] vs. [40, 65]	1.83	1.14–2.93	**0.012**	1.84	1.14–2.96	**0.012**
Gender (M vs. F)	1.03	0.72–1.46	0.882	1.03	0.72–1.46	0.891
Smoking (ever vs. never)	0.73	0.52–1.03	0.076	0.70	0.50–1.00	0.051
Familiarity for PD or tremor	4.23	2.34–7.65	** <0.001**	4.26	2.34–7.75	** <0.001**
Born in the BS Province	1.70	1.00–2.87	**0.048**	1.71	1.01–2.89	**0.047**
Born in a farm	1.77	1.10–2.85	**0.018**	1.79	1.11–2.89	**0.017**
Being exposed to metals	2.37	1.34–4.21	**0.003**	1.85	0.70–4.86	0.212
*SNCA* rs356219 (TC vs. TT)	1.24	0.87–1.78	0.230	1.20	0.82–1.74	0.347
*SNCA* rs356219 (CC vs. TT)	2.03	1.25–3.30	**0.004**	1.93	1.15–3.21	**0.012**
Exposed to metals^*^SNCA (TC vs. TT)				1.39	0.40–4.85	0.603
Exposed to metals^*^SNCA (CC vs. TT)				1.63	0.29–9.03	0.575
**Parkinsonism**						
Age (q.les) (65,72] vs. [40, 65]	2.75	1.20–6.34	**0.017**			
Age (q.les) (72,78] vs. [40, 65]	4.22	1.84–9.71	**0.001**			
Age (q.les) (78,97] vs. [40, 65]	4.97	2.13–11.59	** <0.001**			
Gender (M vs. F)	1.12	0.64–1.95	0.693			
Smoking (ever vs. never)	1.12	0.65–1.91	0.690			
Familiarity for PD or tremor	4.30	1.94–9.52	** <0.001**			
Born in the BS Province	2.27	0.86–5.99	0.099			
Born in a farm	2.63	1.40–4.95	**0.003**			
Being exposed to metals	1.36	0.53–3.44	0.521			
*SNCA* rs356219 (TC vs. TT)	1.85	1.05–3.29	**0.035**			
*SNCA* rs356219 (CC vs. TT)	2.48	1.15–5.33	**0.020**			

Adjusted regression odds ratios, 95% CI, and their p-values are shown. p-values below 0.05 are shown in bold. The symbol

* * represents the product between metal exposure and SNCA, for more clarity we can substitute it with x*.

## Discussion

This study highlights the increased risk of PD in relation to exposure to metals and increased risk of Parkinsonism and PD in relation to a *SCNA* polymorphism. The role of genetics is further stressed by the significant effect of positive family history: among the putative risk factors, family history showed the strongest association with Parkinsonism and PD.

This study is consistent with earlier studies about exposure to metals and risk of Parkinsonism and PD where increased risk of PD have been associated with to occupational exposure to Mn, Pb, Cu, and Fe (44) and Fe intake (45) (the first study recruited a total of 608 patients while the second study was a meta-analysis on a total of 126,507 subjects). Further, in the province of Brescia, exposure to Mn has been shown as potentially related to the increased risk of Parkinsonism and PD based on ecological observation (1). We were not able to consider exposure to single metals since for each element we had too few observations for cases and controls. However, this association was mainly driven by the exposure to Pb which was the most frequent element to which the subjects were exposed to and it is also known as the most dangerous for PD among all metals (46). Our results are also consistent with previous studies on the increased risk of PD and Parkinsonism due to pesticide exposure (47–50) that we identified through the fact of being born in a farm.

Discovery of mutations in genes causing inherited forms of PD and epidemiological, *in vitro*, and *in vivo* evidence of environmental risk factors have improved the knowledge of mechanisms underlying PD. Most cases are likely due to different combinations of environmental exposures and genetic susceptibility (35).

*SNCA* is a key gene in the pathophysiology of Parkinsonism/PD and was the first autosomal dominant gene to be identified. Rare missense mutations as well as gene triplications in *SNCA* have been identified as causes for rare familial forms of PD, mediating disease through changes in protein fibrillation or higher expression of the protein, alpha-synuclein (51–53). These mutations account for only a small proportion of PD cases. More common genetic variants, although neither necessary nor sufficient to lead to disease, also have a modest effect on increasing the transcription of *SNCA*. Genome-wide association studies have demonstrated association between *SNCA* common variants and susceptibility to sporadic PD disease (54). In line with our findings, a recent meta-analysis showed that rs356219 is associated with increased risk of PD (55) (see ref in the comment field), while a recent study found that *SNCA* can modulate the PD age at onset (56).

Interactions between Mn and *SNCA* has been studied extensively in *in vivo* and *in vitro* studies (32). The neurotoxicity of *SNCA* and the role of Mn seem to be intensity and time depending (31, 32). *SNCA* may be involved in the regulation of neuronal manganese and actually be neuroprotective against acute manganese exposure (33). Chronic manganese exposure has been shown to promote alpha-Synuclein aggregation and toxicity in dopaminergic cells (34, 57, 58).

We only observed a marginally significant influence (but with high uncertainty due to the wide CI) from polymorphism in *ATP13A2* (also known as *PARK9*) rs4920608 on the risk of developing PD and Parkinsonism. The same polymorphisms that were assessed here have shown gene-environment interactions with Mn on Parkinsonism in other studies (25). A recessive mutation in the ATP13A2 encoding for the lysosomal P-type ATPase “PARK9” causes the inherited parkinsonian syndromes ‘Parkinson's disease 9′ (or Kufor-Rakeb syndrome, PARK9, #606693). Loss of ATP13A2 function in mice causes sensorimotor deficits, increased sensitivity to manganese and accumulation of alpha-synuclein (35).

No associations were observed between polymorphisms in *SLC30A10* or *SLC39A8* and PD or Parkinsonism in this study. *SLC30A10* and *SLC39A8* are both involved in Mn transport (59, 60) and polymorphisms in these genes have been associated with blood Mn concentrations (27, 60, 61) and linked to neurological outcomes (27, 62, 63) but so far not to PD. Lack of evidence of statistical interaction in this, as well as other studies, may be due to the low frequencies of subjects exposed to metals and in each polymorphism variant and does not rule out biological interaction (64). One should also be aware that statistical interaction depends upon the statistical model used to represent the relationships between the risk factors and the disease and that its representation is scale-dependent. In a generalized linear model interaction is defined as a deviation from additivity of the effects of the risk factors, measured in the scale of the linear predictor. According to an inherently multiplicative model, as the logistic regression model is, the contemporaneous presence of genetic and environmental risk factors produces a multiplication of the risk, calculated on an OR scale. Furthermore, as this is a case-control study, this study has the disadvantage of recall bias. While case-control studies are ideal to study disease with a long latency period, such as Parkinsonism/PD, participants in this study may have been subjected to recall bias when asked about metal exposure. We enrolled considerable number of subjects who participated with the assistance of a caregiver especially among cases (30.5% while 9.2% among controls), however only 5.3% cases and 1.6% controls needed full caregivers' assistance. To minimize the recall bias, we conducted direct face-to-face interviews in the participating hospitals. In addition, the measurement for metal exposure was a crude yes or no. Lack of a precise measurement among participants may have resulted in non-differential misclassification of exposure, which could have biased the results toward the null. In addition, there may be other biological factors that are playing a role in the interaction between genetics and the outcome of Parkinsonism and PD that are not yet identified. The strength of this study is the homogeneity of the study population as well as the completion of data on a large set of known risk factors for a uniquely susceptible population with a rare exposure.

Through this work we were able to assess that lifetime exposure to metals and genetic predisposition in *SNCA* are determinants of PD and parkinsonism in the highly industrialized area of Brescia, Italy.

## Data Availability Statement

The raw data supporting the conclusions of this article will be made available by the authors, without undue reservation.

## Ethics Statement

The studies involving human participants were reviewed and approved by Ethical Committee of the Spedali Civili, Brescia. The patients/participants provided their written informed consent to participate in this study.

## Author Contributions

RL: overall coordination. SG, SR, and DH: statistical analysis. KB, LC, CP, and KW: genetic measurement/interpretation. MC, PC, MO, FP, MR, and MT: data collection. UG: epidemiological study design. DH, APi, and APa: paper revision/interpretation. DP: occupational history coding and study coordinator. All authors contributed to the article and approved the submitted version.

## Conflict of Interest

The authors declare that the research was conducted in the absence of any commercial or financial relationships that could be construed as a potential conflict of interest.
