# Supplementary material for: Metal Exposure and SNCA rs356219 Polymorphism Associated With Parkinson Disease and Parkinsonism
Source: Front Neurol. 2020 Dec 9;11:556337. doi: 10.3389/fneur.2020.556337 (PMC7755861; doi:10.3389/fneur.2020.556337)
Supplement: Supplementary file 1 [file Data_Sheet_1.docx]

Supplementary Material

**Supplementary Table 1**: Effect of the *SNCA* rs356219 according to codominant, dominant and recessive models.

|  | Control | % | Case | % | OR | lower | upper | p-value | AIC |
| --- | --- | --- | --- | --- | --- | --- | --- | --- | --- |
| Codominant |  |  |  |  |  |  |  |  |  |
| T/T | 181 | 42.6 | 141 | 34.1 | 1.00 |  |  | 0.0112 | 1160.0 |
| T/C | 190 | 44.7 | 196 | 47.3 | 1.32 | 0.98 | 1.78 |  |  |
| C/C | 54 | 12.7 | 77 | 18.6 | 1.83 | 1.21 | 2.76 |  |  |
| Dominant |  |  |  |  |  |  |  |  |  |
| T/T | 181 | 42.6 | 141 | 34.1 | 1.00 |  |  | 0.0110 | 1160.5 |
| T/C-C/C | 244 | 57.4 | 273 | 65.9 | 1.44 | 1.09 | 1.90 |  |  |
| Recessive |  |  |  |  |  |  |  |  |  |
| T/T-T/C | 371 | 87.3 | 337 | 81.4 | 1.00 |  |  | 0.0185 | 1161.4 |
| C/C | 54 | 12.7 | 77 | 18.6 | 1.57 | 1.08 | 2.29 |  |  |

**Supplementary Figure 1**: Kaplan-Meier estimates of the cumulative proportion of cases as a function of the age at PD onset for the three *SNCA* rs356219 genotypes


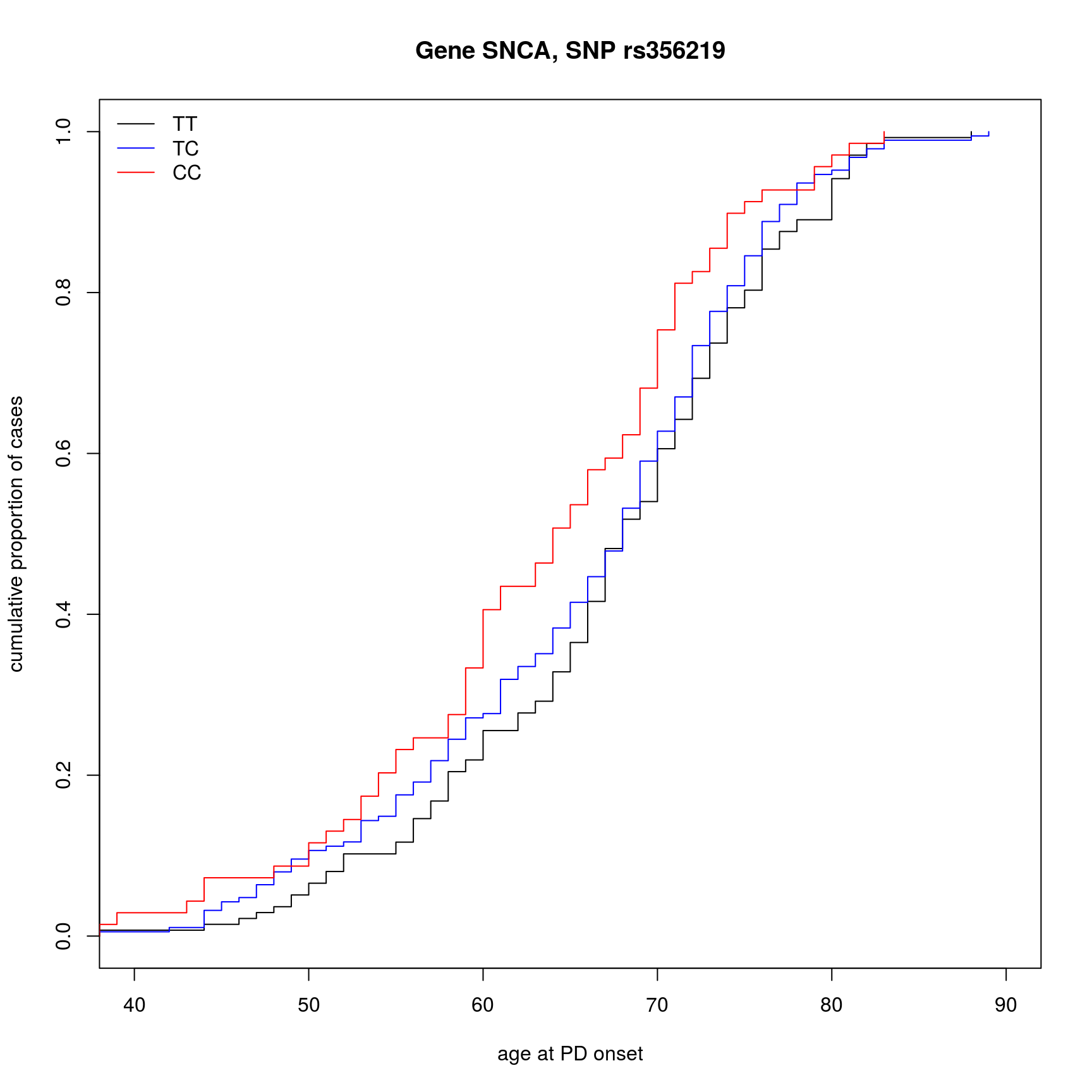


**Supplementary Table 2**: Questions included in the questionnaire to collect information related to occupational metal exposures.

| Have you ever had a job in which you came in contact with Aluminum? |
| --- |
| Have you ever had a job in which you came in contact with Antimony? |
| Have you ever had a job in which you came in contact with Arsenic? |
| Have you ever had a job in which you came in contact with Beryllium? |
| Have you ever had a job in which you came in contact with Cadmium? |
| Have you ever had a job in which you came in contact with Copper? |
| Have you ever had a job in which you came in contact with Chromium? |
| Have you ever had a job in which you came in contact with Gallium? |
| Have you ever had a job in which you came in contact with Magnesium? |
| Have you ever had a job in which you came in contact with Manganese? |
| Have you ever had a job in which you came in contact with Mercury? |
| Have you ever had a job in which you came in contact with Nickel? |
| Have you ever had a job in which you came in contact with Lead? |
| Aluminum [starting year] |
| Aluminum [ending year] |
| Antimony [starting year] |
| Antimony [ending year] |
| Arsenic [starting year] |
| Arsenic [ending year] |
| Beryllium [starting year] |
| Beryllium [ending year] |
| Cadmium [starting year] |
| Cadmium [ending year] |
| Copper [starting year] |
| Copper [ending year] |
| Chromium [starting year] |
| Chromium [ending year] |
| Gallium [starting year] |
| Gallium [ending year] |
| Magnesium [starting year] |
| Magnesium [ending year] |
| Manganese [starting year] |
| Manganese [ending year] |
| Mercury [starting year] |
| Mercury [ending year] |
| Nickel [starting year] |
| Nickel [ending year] |
| Lead [starting year] |
| Lead [ending year] |
